# A conserved cluster of three PRD-class homeobox genes (*homeobrain*, *rx *and *orthopedia*) in the Cnidaria and Protostomia

**DOI:** 10.1186/2041-9139-1-3

**Published:** 2010-07-05

**Authors:** Maureen E Mazza, Kevin Pang, Adam M Reitzel, Mark Q Martindale, John R Finnerty

**Affiliations:** 1 Department of Biology, Boston University, 5 Cummington Street, Boston, MA 02215, USA; 2 Kewalo Marine Lab, Pacific Biosciences Research Center, University of Hawaii, 41 Ahui St., Honolulu, HI 96813, USA; 3 Woods Hole Oceanographic Institution, Woods Hole, MA 02543, USA

## Abstract

**Background:**

Homeobox genes are a superclass of transcription factors with diverse developmental regulatory functions, which are found in plants, fungi and animals. In animals, several Antennapedia (ANTP)-class homeobox genes reside in extremely ancient gene clusters (for example, the Hox, ParaHox, and NKL clusters) and the evolution of these clusters has been implicated in the morphological diversification of animal bodyplans. By contrast, similarly ancient gene clusters have not been reported among the other classes of homeobox genes (that is, the LIM, POU, PRD and SIX classes).

**Results:**

Using a combination of *in silico *queries and phylogenetic analyses, we found that a cluster of three PRD-class homeobox genes (*Homeobrain (hbn)*, *Rax (rx) *and *Orthopedia (otp)*) is present in cnidarians, insects and mollusks (a partial cluster comprising hbn and rx is present in the placozoan *Trichoplax adhaerens*). We failed to identify this 'HRO' cluster in deuterostomes; in fact, the *Homeobrain *gene appears to be missing from the chordate genomes we examined, although it is present in hemichordates and echinoderms. To illuminate the ancestral organization and function of this ancient cluster, we mapped the constituent genes against the assembled genome of a model cnidarian, the sea anemone *Nematostella vectensis*, and characterized their spatiotemporal expression using *in situ *hybridization. In *N. vectensis*, these genes reside in a span of 33 kb with the same gene order as previously reported in insects. Comparisons of genomic sequences and expressed sequence tags revealed the presence of alternative transcripts of Nv-otp and two highly unusual protein-coding polymorphisms in the terminal helix of the Nv-rx homeodomain. A population genetic survey revealed the Rx polymorphisms to be widespread in natural populations. During larval development, all three genes are expressed in the ectoderm, in non-overlapping territories along the oral-aboral axis, with distinct temporal expression.

**Conclusion:**

We report the first evidence for a PRD-class homeobox cluster that appears to have been conserved since the time of the cnidarian-bilaterian ancestor, and possibly even earlier, given the presence of a partial cluster in the placozoan *Trichoplax*. Very similar clusters comprising these three genes exist in *Nematostella *and diverse protostomes. Interestingly, in chordates, one member of the ancestral cluster (*homeobrain*) has apparently been lost, and there is no linkage between *rx *and *orthopedia *in any of the vertebrates. In *Nematostella*, the spatial expression of these three genes along the body column is not colinear with their physical order in the cluster but the temporal expression is, therefore, using the terminology that has been applied to the Hox cluster genes, the HRO cluster would appear to exhibit temporal but not spatial colinearity. It remains to be seen whether the mechanisms responsible for the evolutionary conservation of the HRO cluster are the same mechanisms responsible for cohesion of the Hox cluster and other ANTP-class homeobox clusters that have been widely conserved throughout animal evolution.

## Background

Gene clusters have been crucially important in the evolution of animals because close physical linkage affects genetic recombination, molecular evolution and gene regulation [1-7]. For example, clustering can contribute to coordinated transcriptional regulation of linked genes if the local chromatin structure affects several genes in the same chromosomal neighborhood or if shared regulatory elements drive the expression of neighboring genes, such as the gene clusters of the human cardiac transcriptome [8].

Gene clusters are also important tools for reconstructing genome evolution. The clustering of related genes can reveal the mechanism underlying the expansion of a gene family, and conserved gene clusters define homologous chromosomal segments that may be compared across species [9-21].

Homeobox gene clusters, in particular, have received a great deal of study. Since the pioneering studies by Ed Lewis and co-workers on the Antennapedia (ANTP) and Bithorax complexes of *Drosophila *[22], comparative genomic studies have revealed (i) that certain homeobox clusters are widely conserved throughout the animal kingdom, (ii) that clustering may influence gene expression and (iii) that clustering may be conserved by stabilizing selection if disrupting the cluster has deleterious effects on the spatiotemporal expression of linked genes [23-28].

All of the aforementioned homeobox clusters consist of *ANTP*-class genes [29]. Based on published studies, conserved clusters of non-*ANTP *class homeobox genes that date from the time of the bilaterian common ancestor do not appear to be as common, although less ancient clusters of PRD (PRD) class genes have been identified. A cluster of several *Rhox *genes that function during gametogenesis appears to be restricted to rodents [15]. A cluster of three *Otx *genes has been identified in the sea anemone *Nematostella*, but this cluster appears to be restricted to anthozoan cnidarians [30].

The widespread occurrence of conserved ANTP-class clusters could reflect the unique early history of this class; recent studies suggest that the various ANTP-class gene clusters can all be traced to four ancient arrays (extended Hox, ParaHox, NKL and EHG box [10]) or even to a single ancestral 'metaHox' cluster [9]. Different elements of the ancestral linkage arrangements could have been conserved in different animal lineages. The non-ANTP homeobox genes may never have been organized in metaclusters, so the retention of subclusters would not have been possible. Alternatively, the apparent paucity of evolutionarily conserved non-ANTP homeobox clusters may simply reflect a search bias; the effort to identify clusters of Hox-related genes has spanned about 20 years and has involved dozens of laboratories. A comparable effort to identify clusters of non-ANTP homeobox genes has not yet been undertaken; however, conserved clusters of Iroquois (*irx*) genes have been identified in arthropods and vertebrates [21]. The insect-crustacean ancestor is thought to have possessed a cluster of two *irx *genes, whereas a cluster of three *irx *genes is thought to have been present in the common ancestor of vertebrates [21]. It is not yet clear whether a cluster of *irx *genes was already present in the protostome-deuterostome ancestor, or whether clusters arose independently in arthropods and vertebrates.

A cluster of three PRD-class homeobox genes (*Homeobrain (hbn)*, *Rax (rx) *and *Orthopedia (otp)*) has been reported in *Drosophila *[31]. These three genes are clustered within a span of ~38 kb on chromosome 2, and they exhibit very similar spatiotemporal expression. All three genes are involved in patterning specific regions of the embryonic brain. To date, a comparable cluster has not yet been described in other species.

In this report, we describe the *hbn*-*rx*-*otp *cluster in the sea anemone, *Nematostella vectensis*. The *Nematostella *cluster spans only ~33.5 kb and the relative order of the genes is the same as in *Drosophila*, although the transcriptional orientation of *orthopedia *is reversed. We also report the conservation of all or part of this cluster in other protostomes including a mollusk and a number of insects (for example, mosquito, honeybee and flour beetle), suggesting that (i) this cluster was present in the cnidarian-bilaterian ancestor and (ii) linkage between these genes may be under strong stabilizing selection in some lineages. In the sea anemone, *in situ *hybridization reveals that all three genes are expressed in the ectoderm in non-overlapping territories along the oral-aboral axis. *NvHbn *is expressed during early gastrula stages around the blastopore (future mouth), and expression persists in the ectoderm around the base of the tentacles into the juvenile polyp stage. *NvRx *is expressed in individual cells in the aboral ectoderm, and is first expressed in mid gastrula stages. *NvOtp *expression first becomes apparent in the larva, in the ectodermal layer of the pharynx. Thus, in *Nematostella*, the expression of these clustered genes is consistent with temporal but not spatial colinearity.

## Materials and methods

### Retrieval of *Nematostella *PRD-class homeodomain genes

In total, 33 PRD-class homeobox genes were previously identified in the *Nematostella *genome [32] by conducting BLAST searches of a draft genome assembly (available at StellaBase; http://www.stellabase.org/[33]). Phylogenetic analysis of homeodomain sequences by Bayesian and neighbor-joining methods identified single representatives of the *homeobrain *(HBN_Nv079), *orthopedia *(OTP_Nv047) and *rx *(RX_Nv129) families in *Nematostella *[32]. Subsequently, we designed gene-specific primers to amplify the 3' and 5' ends of the *hbn*, *otp *and *rx *transcripts from cDNA (Table 1). Overlapping 5' and 3' RACE (rapid amplification of cDNA ends) fragments were conceptually spliced to reconstruct complete transcripts. In addition, *hbn*, *otp *and *rx *transcripts were also identified among 150,000 *Nematostella *expressed sequence tags (ESTs) sequenced as part of the genome-sequencing project recently completed by the Joint Genome Institute [34]. Once the full-length transcripts were assembled, the longest open reading frames (ORFs) in frame with the highly conserved homeodomain were inferred using MacVector V.7.2.3 (Accelrys Inc., San Diego, CA, USA).

**Table 1:** Primers used to amplify RACE products and characterize Rx polymorphism.

Gene	Procedure	Name	Sequence 5' → 3'
*hb*	5'-RACE	F1	CAGGCGAAGTCGAACTACGTTCACGACATACC
		F2	CGCAA-TACCCCGATGTGTTCACGAGAGAGG
	3'-RACE	R1	TCTTGCCTCGCT-CAAGTCCAGCCGAAG
		R2	GGGCAAGTTCCTCTCTCGTGAACACATCGG
*otp*	5'-RACE	R1	CGCTGCCAGCTCTTCTCGCATGAACAC
		R2	CGGA-TAGTGAGTCCTGGCGAAACATCGC
	3'-RACE	F1	CGAGCGATGTTT-CGCCAGGACTCACTATC
		F2	CGGATGTGTTCATGCGAGAAGAGCTGG
*rx*	5'-RACE	R1	TGTACCCTAACTTCAGGGAGGCTTATTTTTAGCG
		R2	TGGATAGTGGGATTTCTCGAAGGCTCGC
	3'-RACE	F1	TCAGAAGA-AACCGTACCACCTTCACAACG
		F2	TCGAGCGAGCCTTCGAGAAATCCC
	polymorphism	RX_for	GATCCTTAATTCAAACCCTGGGAC
		RX_rev	TCGGGCAAATCTGA TACGAGTAG

RACE = rapid amplification of cDNA ends.

### Retrieval of PRD-class homeodomains from other taxa

Human and fruit fly (*Drosophila melanogaster*) homeodomains from the PRD class were taken from a previous study [32]. We searched for *hbn*, *rx *and *otp *homeodomains in the partially or wholly sequenced genomes of four additional vertebrates (*Mus musculus*, *Gallus gallus*, *Xenopus tropicalis *and *Danio rerio*), four non-vertebrate deuterostomes (*Ciona intestinalis*, *Branchiostoma floridae*, *Saccoglossus kowalevskii *and *Strongylocentrotus purpuratus*), three additional insects (*Anopheles gambiae*, *Apis mellifera *and *Tribolium castaneum*), one nematode (*Caenorhabditis elegans*), one mollusk (*Lottia gigantea*), one annelid (*Capitella teleta*) and a placozoan (*Trichoplax adhaerens*). Database searches were conducted via Entrez http://www.ncbi.nlm.nih.gov/gquery/gquery.fcgi using gene names (*orthopedia*, *rx*/*rax*, *homeobrain*), and similarity searches were performed using the tBLASTn and BLASTp search algorithms. The homeodomains of *hbn*, *otp *and *rx *from *Nematostella*, human and fruit fly were used as the query sequences. Sequences that matched the query sequence were then subjected to reciprocal tBLASTn and/or reciprocal BLASTp similarity searches against the genome from which the query sequence was derived. We formally tested for orthology with phylogenetic analyses (see below).

### Determination of gene linkage and gene structure

Predicted homeobox sequences for *Nematostella hbn*, *otp *and *rx *were mapped to the publicly available assembly of the *Nematostella *genome (DOE-Joint Genome Institute (JGI) *Nematostella vectensis *genome assembly 1.0; http://genome.jgi-psf.org/Nemve1/Nemve1.home.html) using BLASTn to determine whether the *hbn*, *rx *and *otp *loci of *Nematostella *are linked as in *Drosophila *[31]. Gene structures were determined by mapping transcripts for *hbn*, *rx *and *otp *against the genome. To identify possible non-homeodomain genes that might be closely linked to *hbn*, *rx *and *otp*, the regions flanking these genes were compared against the RefSeq database using BLASTx (V.2.2.15).

### Identification of homeobrain-rx-orthopedia clusters in other taxa

The genomic organization of the *hbn*, *rx *and *otp *cluster in *Drosophila *has been described previously [31]. In other species in which two or three of these PRD-class genes were identified, the genes were mapped to the corresponding assembled genome to determine if they might be linked. The level of coverage for different assemblies varies widely, from completed genome assemblies for human, mouse, fruit fly and nematode, to assemblies at the level of scaffolds, contigs and linkage groups for numerous other animals. Therefore, we have uncertainty for linkage of some genes that are present in the genome because current assemblies limit absolute characterization of location. For species in which at least two of the three genes matched the same scaffold, linkage group or chromosome, we determined the relative position and transcriptional direction of each gene from the cluster.

### Phylogenetic analysis

**Figure 1:**
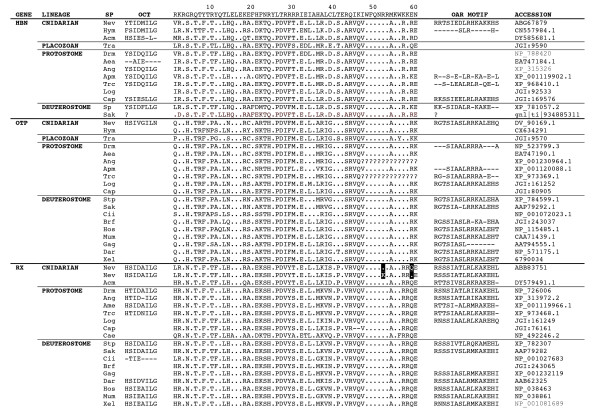
**Alignment of octapeptide regions, homeodomains and OAR domains from representative *hbn*, *otp *and *rx *genes of cnidarians, protostomes and deuterostomes**. Homeodomain sequences are aligned against the *Drosophila antennapedia *homeodomain. *Nematostella*, human and fruit fly representatives were taken from Ryan *et al. *[32]. Homeodomains from additional taxa were identified by similarity searches (tBLASTn and BLASTp) conducted through the BLAST interface at National Center for Biotechnology Information (see Methods) and at respective genome assemblies available through the Joint Genome Institute. Abbreviations for taxa are as follows: (cnidarians: *Acropora millepora *(Acm), *Hydra magnipapillata *(Hym), *Nematostella vectensis *(Nev); placozoan: *Trichoplax adhaerens *(Tra); protostome bilaterians: *Aedes aegypti *(Aea), *Anopheles gambiae *(Ang), *Apis mellifera *(Apm), *Caenorhabditis elegans *(Cae), *Capitella teleta *(Cap), *Lottia gigantea *(Log), *Tribolium castaneum *(Trc); deuterostome bilaterians: *Branchiostoma floridae *(Brf), *Ciona intestinalis *(Cii), *Danio rerio *(Dar), *Gallus gallus *(Gag), *Homo sapiens *(Hos), *Mus musculus *(Mum), *Saccoglossus kowalevskii *(Sak), *Strongylocentrotus purpuratus *(Stp), *Xenopus laevis *(Xel)). The octapeptide [72,73] and/or the OAR domain [51,75] are indicated when identified. Two different versions of the *Rx *homeodomain of *Nematostella *are shown, and the polymorphic residues are highlighted.

*Hbn*, *otp*, *rx *and other PRD-class homeodomain sequences were aligned using a web implementation of the computer program Muscle [35]. The default parameters were used, and the alignment algorithm did not introduce any alignment gaps. Phylogenetic analysis was performed using the neighbor-joining method as implemented in the computer package Phylip (V.3.61; [36]). In total, 111 PRD-class homeodomains were used in the phylogenetic analysis, including 33 from *Nematostella*, 39 from human and 24 from fruit fly, which had been identified in a previous study [32]. Human and fruit fly homeodomain families comprising multiple invariant or nearly invariant homeodomains were pared to a single representative. Select homeodomains were added to provide broader representation from deuterostomes and protostomes (Deuterostomia: *Branchiostoma floridae *(*otp*, *rx*), *Strongylocentrotus purpuratus *(*otp*, *rx*, *hbn*), *Saccoglossus kowalevskii *(*otp*, rx); Protostomia: *Caenorhabditis elegans *(*rx*), *Patella vulgata *(*otp*), *Platynereis dumerilii *(*rx*) and *Capitella teleta *(*hbn*). *Distalless *and *hox1 *homeodomains from *Drosophila melanogaster *and *Branchiostoma floridae *were included as outgroups. Homeodomain sequences and accession numbers for *homeobrain*, *otp *and *rx *are provided in Figure 1. Sequences and accession numbers for the other PRD-class homeodomains used in the phylogenetic analysis are available from Ryan *et al. *[32]. Pairwise distances between homeodomains were calculated using the PAM distance matrix. Support for individual nodes was assessed using 1,000 replicates of the bootstrap [36]. Amino acid substitutions were localized to particular branches on the phylogeny using MacClade V.4 [37].

### Population genetic analysis of polymorphisms in the homeodomain of Rx

To confirm the existence and determine the geographic distribution of two unusual polymorphisms at positions 52 and 59 within the homeodomain of *Nematostella Rx*, we designed primers to amplify a 585 bp fragment of the gene (Table 1). PCR was carried out using 0.5 U Taq DNA polymerase, 1 × Mg-free PCR buffer, 1.7 mM MgCl_2_, 0.67 mM dNTPs, 1 μm of each primer and approximately 5 ng genomic DNA in 20 μl reactions. Thermal cycling consisted of denaturation for 5 minutes at 94LC, followed by 30 cycles of 30 seconds at 94°C, 30 seconds at 60°C and 1 minute at 72°C, with a final extension of 15 minutes at 72°C. PCR products were directly sequenced (RX_for; Macrogen, Seoul, South Korea). All polymorphisms were verified by eye.

### *In situ *hybridization

Digoxygenin-labeled riboprobes were generated from 3' RACE products for each gene (*homeobrain*, 749 nt; *orthopedia*, 1556 nt; *rx*, 1419 nt). Each probe included a portion of the homeobox, plus a region of highly divergent coding sequence downstream of the homeobox and the 3' untranslated region (UTR). Whole-mount *in situ *hybridization was performed on embryos and larvae using published protocols [38]. Stringent hybridization conditions were employed (65°C for 20-44 hours using probe concentrations of 1.0 ng/μl).

## Results

### *Nematostella *Otp, Rx and Hbn transcripts and gene structure

Using RACE PCR, we cloned and annotated full transcripts for *otp*, *rx *and *hbn *from *Nematostella *(accession numbers HM004556-8). In addition, we queried the EST databases for *Nematostella *to further annotate these genes and to identify non-synonymous polymorphisms in each gene.

### Otp

The *otp *transcript is 1901 nucleotides long, and encodes a predicted protein of 291 amino acids. When mapped to the genomic sequence, this transcript spans 9587 nucleotides, including four exons (see Additional file 1). We identified two putative full-length *otp *transcripts that encode different splice variants in *Nematostella*. Comparing the two splice variants of the *Nematostella otp *gene, one lacked a highly conserved transcriptional repression domain that was present in the other and that is typical of *otp *orthologs from other taxa. Neither splice variant that we have identified encodes an OAR (*otp*, *aristaless *and *rax*) domain. However, a highly conserved version of this domain is predicted to be encoded by a stretch of nucleotides that is located immediately downstream and in frame with exon 3 (see Additional file 1, boxed sequence). The predicted OAR motif of *Nematostella Otp *is highly conserved relative to deuterostome sequences; in fact, it is identical to both the human and sea urchin sequences at 15 of 16 residues. By contrast, in protostome *orthopedia *proteins, only the central core of the OAR domain appears to be conserved (for example, SIAALRRRA in *Drosophila*), or the OAR domain appears to have been lost entirely (for example, in *Aedes aegypti *or *Anopheles gambiae*). Given this degree of sequence conservation, we predict that *Nematostella *expresses at least one additional *Otp *splice variant that includes this OAR domain. Whereas other reported *orthopedia *proteins lack an octapeptide domain upstream of the homeodomain, the *Nematostella *gene encodes a stretch of eight residues upstream of the homeodomain that shares five out of eight residues with the octapeptide of deuterostome Rx proteins (HSIxxILx; Figure 1).

### Rx

The *Nematostella rx *transcript we assembled using RACE is 1792 nucleotides long and it encodes a predicted protein 266 amino acids long (see Additional file 2). It extends over a 4972-nucleotide region of the genome, comprising three exons (see Additional file 2). There are three noteworthy regions of similarity between the *rx *proteins of *Nematostella *and bilaterians: the homeodomain, the octapeptide and the OAR domain (Figure 1). In the homeodomain, *Nematostella *is identical to both human and fruit fly at 90% of residues (54/60). Among PRD-class homeodomains, the presence of a valine at residue 43 appears to be a synapomorphy of the *rx *family, and this trait is shared by all the *rx *sequences in our dataset. The *rx *family is also characterized by arginine-alanine at positions 18-19 of the homeodomain. This is seen in all taxa except the coral *Acropora millepora*, which has a glutamine at position 18 [29,32,39,40]. The sea anemone is also identical to both human and fruit fly at six of seven residues in the octapeptide. Within the OAR domain, *Nematostella *and human are identical at 13 of 16 residues, and *Nematostella *and *Drosophila *are identical at 14 of 16 residues (Figure 1).

We identified four additional EST sequences for *Nematostella rx*, two that were previously deposited at the National Center for Biotechnology Information (NCBI) (CAGN10625 and DV088198) and two that were generated by the Joint Genome Institute (JGI) as part of the *Nematostella *genome sequencing initiative (2664141-1 and 2664141-2). One of the EST sequences (DV088198) does not encode the complete OAR motif, instead producing a predicted protein 24 residues shorter than the other transcripts (see Additional file 2). Comparison of the genome assembly, our RACE product and these four ESTs revealed 28 single-nucleotide polymorphisms, including six that result in a change to the protein sequence (Additional file 2). Two of the polymorphic amino acid positions reside within helix 3 of the homeodomain, the so-called 'DNA-binding helix' (Figure 1; see Additional file 2): an A/G polymorphism that results in an amino acid change from arginine (R) to lysine (K) at position 52, and a C/G polymorphism results in an amino acid substitution from glutamine (Q) to glutamic acid (E) at position 59, an amide to acidic amino acid. Both the possession of lysine at position 52 and glutamic acid at position 59 are unique within the *Rx *family (Figure 1). In fact, the possession of a lysine at position 52 is very unusual for PRD-class homeodomains in general; of the 111 PRD-class homeodomains included in our phylogenetic analysis, the only other sequence with a lysine at this position is the *Nematostella DMBXd *homeodomain. Over the entire PRD class, the possession of glutamic acid at position 59 is not as uncommon. The Pax4/6 family exhibits glutamic acid at this position, as do three other *Nematostella *PRD-class homeodomains (DMBXb, NVHD_101 and NVHD_148).

To verify the existence of the two nonsynonymous polymorphisms identified in EST sequences (see above) in natural populations and to begin characterizing their geographic distribution, we sequenced a fragment of the *rx *gene from 95 individual animals collected throughout the range of the species (see Additional file 3). At position 52, the phylogenetically unusual lysine variant is relatively rare, accounting for only 15.16% of all alleles, but it does exhibit a broad geographic distribution, being found in seven different estuaries from both the Atlantic and Pacific coasts of North America. However, despite the wide distribution of the K allele, KK homozygotes were only recovered in a single location, Kingsport in Nova Scotia. The apparently limited geographic distribution of this genotype is interesting considering that KR heterozygotes accounted for more than a quarter of all individual animals assayed (RR = 71.58%, RK = 26.16%, KK = 2.11%). At position 59, the glutamine variant accounted for 96.28% of all alleles. Despite its overall rarity (3.72%), the glutamic acid variant was recovered in four widely separated estuaries in New Jersey, Maryland, California and Washington, although no EE homozygotes were recovered in any individual (QQ = 92.55%, QE = 7.45%, EE = 0.00%).

### Hbn

The *Nematostella homeobrain *transcript that we assembled using RACE encompasses three exons (see Additional file 4). Similar to *otp *and *rx*, an intron interrupts the homeodomain between positions 46 and 47. The third exon, 647 nucleotides in length, encodes the final 14 amino acids of the homeodomain plus an OAR motif, which is located near the carboxy terminus of the predicted protein. The *Nematostella homeobrain *protein has high similarity to insect orthologs within the homeodomain region but very little similarity could be detected elsewhere. For example, the homeodomain is identical to its *Drosophila *ortholog at 95% of residues (57/60). Interestingly, the sequence identity between *Nematostella *and *Drosophila *exceeds the sequence identity between *Nematostella *and its fellow cnidarian, *Hydra magnipapillata *(83%; 53 of 60 identical residues). This suggests that the *Hydra *homeodomain has evolved relatively quickly.

### Phylogenetic analysis

**Figure 2:**
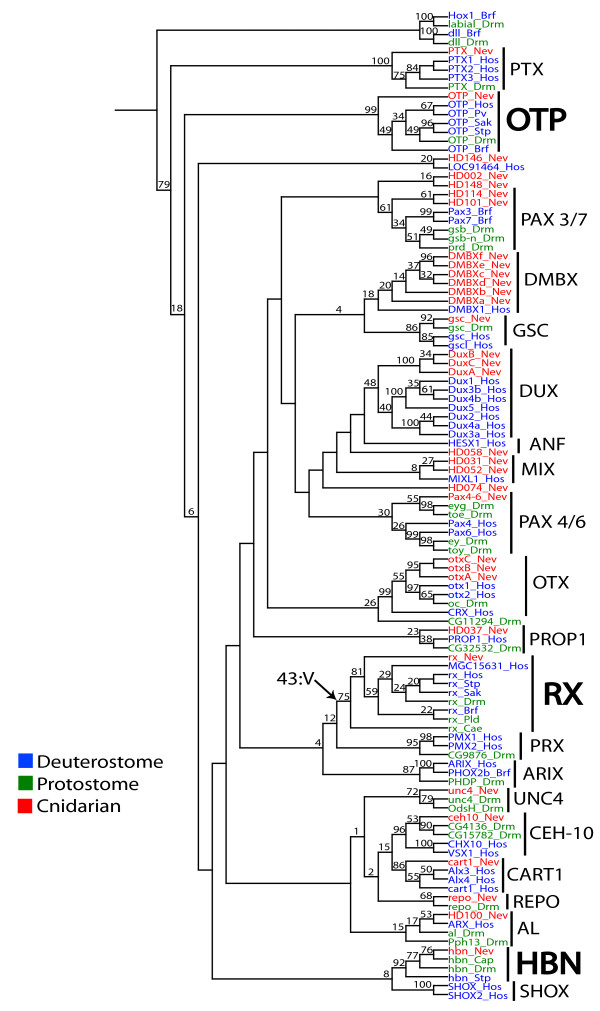
**Neighbor-joining phylogeny of *PRD *class genes based on homeodomain sequences**. Pairwise distances between homeodomains were calculated using the PAM matrix in the computer program protdist in the Phylip package (Phylogenetic Inference Package, V.3.61). Abbreviations for taxa are as in Figure 1. Numbers above nodes indicate the percentage of 1000 bootstrap replicates in which the given node was recovered in a neighbor-joining analysis. The tree is rooted using *Hox1 *and *distalless *homeodomains from the lancelet and the fruit fly.

The maximum-likelihood (ML) and neighbor-joining (NJ) analyses performed here reinforce the conclusions of Ryan *et al. *[32]: *Nematostella hbn*, *otp *and *rx *sequences group with putative orthologs from bilaterian taxa with a moderate to high level of bootstrap support (Figure 2). The *Nematostella hbn *homeodomain is nested within a clade that also includes *hbn *sequences from multiple animals including protostomes (*for example*, *Capitella *and *Drosophila*), *Trichoplax *and two deuterostomes (*Strongylocentrotus *and *Saccoglossus*). Bootstrap support for this clade is moderate (ML = 77, NJ = 64). We were unable to recover a clear *hbn *ortholog from any chordate model system [41] . Our phylogenetic analyses recovered a monophyletic grouping for predicted *rx *orthologs from all species sampled except the most basal member queried, the placozoan *Trichoplax*. Similar to the *hbn *clade, bootstrap support was moderate for *rx *orthologs (ML = 43, NJ = 80). Finally, we identified putative *otp *orthologs from all species expect the nematode *C. elegans *(ML = 66, NJ = 98).

### The Homeobrain-Rax-Orthopedia cluster

**Figure 3:**
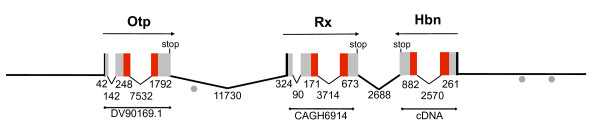
**Organization of the *homeobrain-rx-orthopedia *cluster of *Nematostella***. *Homeobrain *or*thopedia *and *rx *ESTs map to a ~35 kb region within a single scaffold 1,036,593 nucleotides long in the Joint Genome Institute's assembly of the *Nematostella *genome (scaffold 062, Joint Genome Institute *Nematostella vectensis *genome portal, V.1.0). Arrows above the cluster indicate the transcriptional orientation of each gene. The nucleotide length of each intron, exon and intergenic region is indicated below the cluster.

The *Nematostella hbn*, *otp *and *rx *transcripts map to a single scaffold in the publicly available assembly of the *Nematostella *genome (jgi scaffold 62; DOE-JGI *Nematostella vectensis *genome assembly V.1.0; http://genome.jgi-psf.org/Nemve1/Nemve1.home.html). The entire scaffold is 1,036,593 nucleotides long, and the cluster is located roughly 225,000 nucleotides from the nearest end of the scaffold. The *rx *locus intervenes between the *otp *and *hbn *loci. *Otp *and *rx *are encoded on one strand, whereas *hbn *is encoded on the opposite strand (Figure 3). The entire three-gene cluster spans 34,246 nucleotides (from the first nucleotide in the predicted 5' exon of *orthopedia *to the first nucleotide in the predicted 5' exon of *homeobrain*). The distance between the predicted stop codon of *hbn *and the predicted stop codon of *rx *is only 3,437 nucleotides (Figure 3). The intergenic distance between *otp *and the predicted transcription start site of *rx *is 12,158 nucleotides. Using BLASTx we searched for predicted genes positioned in the intergenic regions within the *otp-rx-hbn *cluster. No significant hits were recovered in the smaller intergenic region between *rx *and *hbn *(E < 10). We identified one position of moderate similarity to other proteins in the region between *otp *and *rx *(~ 1 × 10^-6^). Upon closer inspection there were no consistent proteins, regions or domains identified from these results, and the hits appeared as spurious matches. Based on the current assembly of the *Nematostella *genome, the nearest annotated genes bracketing the cluster are a predicted ferredoxin (JGI: 101894) located 16.6 kb from *otp *and a predicted gene similar to RIKEN (JGI: 242384) located 5.8 kb from *hbn*.

**Figure 4:**
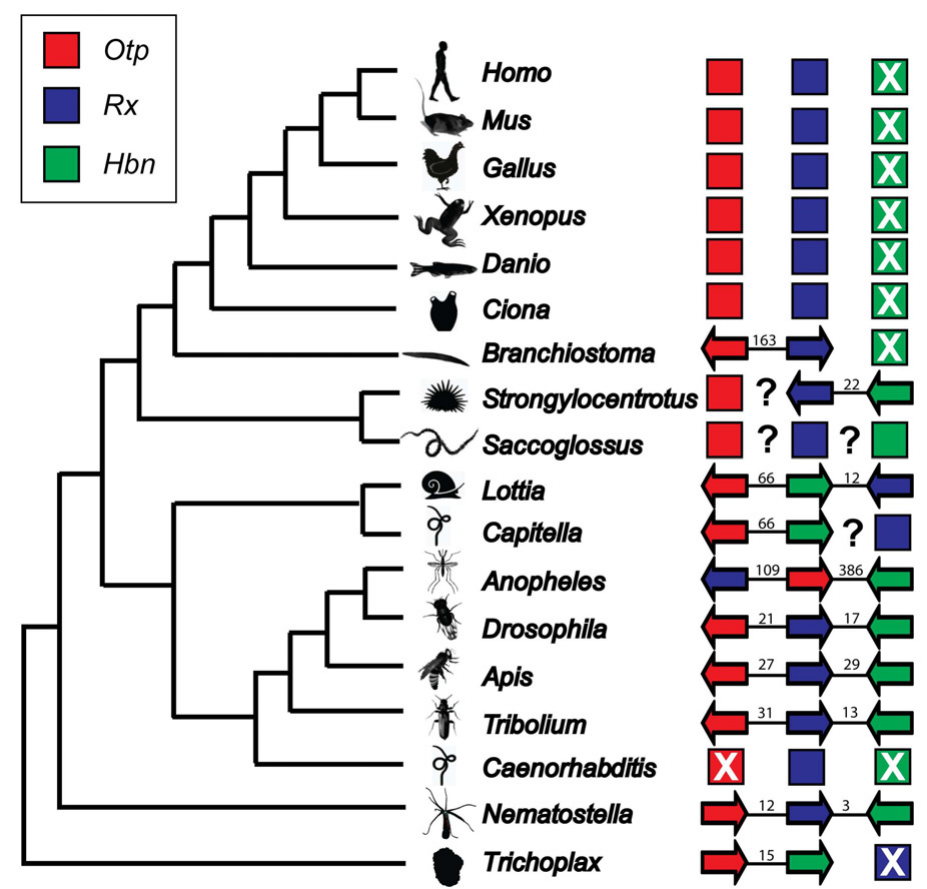
**Phylogenetic distribution of the *Otp*-*Rx*-*Hbn *cluster**. Several sequenced metazoan genomes were searched for evidence of linkage between *orthopedia*, *rx *and *homeobrain*. The genomic organization of the fruit fly homeobrain related gene was previously reported identified by Walldorf *et al. *[31]. Predicted linkage of these three genes was determined for insects (mosquito, honeybee, flour beetle), chordates (vertebrates, amphioxus, ascidian), sea urchin, two lophotrochozoans (limpet, polychaete annelid), sea anemone and placozoan by querying the available genome sequences using the annotated/predicted gene sequences for each gene (this study). Solid lines between genes represent linkage on a single chromosome, scaffold or contig. Approximate distance (kb between genes) is noted above each line, and the relative transcriptional orientation of each gene is indicated by the direction of the arrow (the arrow points from 5' to 3'). Question marks indicate uncertain linkage. Genes confirmed to be on different chromosomes or genomic scaffolds are represented by squares with no interconnecting lines.

The genomic organization of the *Drosophila homeobrain *cluster has been described previously [31]. The fruit fly cluster exhibits the same relative gene order as the *Nematostella *cluster, but the intergenic distances are greater and the transcriptional orientation of *otp *is reversed. The intergenic distance between *hbn *and *rx *is approximately 17.0 kb in the *Drosophila *cluster, whereas the intergenic distance between *rx *and *otp *is approximately 21.0 kb (Figure 4).

Other animal genomes in which *otp*, *rx *and *hbn *genes have been identified or predicted were analyzed for evidence of gene linkage. A *homeobrain *cluster was identified in the sequenced genomes of three additional insects: *Anopheles gambiae *(mosquito), *Apis mellifera *(honeybee) and *Tribolium castaneum *(flour beetle). In honeybee and flour beetle, the cluster organization is similar to that of *Drosophila*: the clusters have the same gene order (*otp*-*rx*-*hbn*), the genes exhibit the same transcriptional orientations and the intergenic distances are comparable (Figure 4). In the mosquito cluster, the gene order and scale are different, with *rx *linked to *otp *across a span of approximately 109 kb, and *otp *linked to *hbn *across a span of approximately 386 kb. In addition, there are a number of genes located in the intergenic spaces between these Paired class genes. For example, a predicted ATP-synthase-like gene is located between *rx *and *otp*, and a calcium/calmodulin protein kinase is located between *otp *and *hbn*. We also identified the three-member *homeobrain *cluster in the limpet *Lottia*. Interestingly, the gene order (*otp*-*hbn*-*rx*) differs from the insects and *Nematostella*, suggesting that an inversion occurred in the lophotrochozoan lineage. For the annelid *Capitella *we were only able to confirm linkage of *otp*-*hbn*, which has a comparable intergenic distance and the same transcriptional orientation as *Lottia*, but *rx *was located on a different scaffold in the current assembly, precluding a confident assessment of linkage. *Rx *is located near the end of the scaffold; however, there is one annotated gene (globin precursor, JGI 21023) between *rx *and the end of scaffold. Furthermore, if the orientation of the *Capitella *cluster is the same as that for *Lottia *(the most closely related species in our analysis), there would be > 400 kb of sequence separating these genes. We identified a number of genes within 40 kb of *hbn*, none of which were *rx *or any homeobox gene.

The sea urchin *Strongylocentrotus purpuratus *and the hemichordate *Saccoglossus kowalevskii *are the only deuterostomes in which we could identify a *homeobrain *gene. Because a public assembly of the *Saccoglossus *genome is not yet available, we were unable to assess linkage and can only report that orthologs for all three members of the cluster are present. In the sea urchin, *hbn *and *rx *are closely linked on the same genomic scaffold (Scaffold_v2_14510, length 454945 nt). The two genes are separated by a distance of approximately 22 kb. This intergenic distance is similar to that observed in *Drosophila*, *Apis*, *Tribolium *and *Lottia*. However, unlike these protostomes, in the urchin, both genes are encoded on the same strand of DNA. *Otp *maps to a different scaffold (Scaffold_v2_10421, length 387609 nt). Because *otp *resides on a different scaffold, evidence of linkage to *hbn *and *rx *could not be established. However, by taking into account the distances flanking *otp *on its scaffold and *hbn *and *rx *on their scaffold, *otp *can be no closer than 437,964 nucleotides from *hbn *and 193,112 nucleotides from *rx*.

We could not identify a *homeobrain *locus in the genomes of the urochordate *Ciona*, the cephalochordate *Branchiostoma *or any of the vertebrate taxa we queried (*Homo*, *Mus*, *Rattus*, *Gallus*, *Xenopus *and *Danio*). In all of the vertebrates and *Ciona*, *otp *and *rx *are located on different chromosomes, so no remnant of the *hbn*-*rx*-*otp *cluster remains. In the current *Branchiostoma *assembly, the *otp *and *rx *homeodomains are separated by ~162 kb on the same genomic scaffold. There are seven annotated genes within the intergenic region (JGI: 102897, 136227, 286088, 102900, 130204, 102902 and 270692) with diverse functions (for example, SCAMP (secretory carrier membrane protein), N-acetyltransferase, tubulin chaperone).

We identified orthologs of *otp *and *hbn*, but not *rx*, in the placozoan *Trichoplax*. Both of these genes are located on the same scaffold, are separated by approximately 15 kb and have the same transcriptional orientation. Because *Trichoplax *represents the earliest diverging taxa in our study, the close proximity of *otp *and *hbn *strongly suggests that these genes have been clustered since very early in animal evolution.

### Developmental expression of otp, rx and homeobrain *Orthopedia*

**Figure 5:**
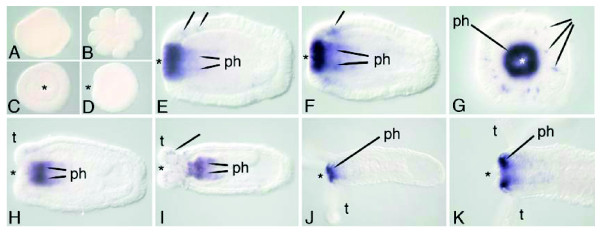
**Expression of *NvOtp***. *In situ *hybridization using a 1556 bp probe of *otp*. In all panels, the asterisk denotes the blastopore or oral end. **(a) **Expression is not detected in the egg, **(b) **during early cleavage or **(c,d) **during gastrulation. **(e,f) **Expression is detected in the planula stage in the ectoderm around the pharynx (ph) and in single ectodermal cells (arrows). **(g) **Oral view of the same planula from (f). **(h,i) **Similar expression in later stages as tentacles (t) begin to grow. **(j,k) **Polyp stages showing high expression in an oral ectodermal ring.

Whole-mount *in situ *hybridization using an antisense *NvOtp *riboprobe of 1,556 bp revealed that expression begins in mid planula stages (Figure 5e), well after gastrulation is complete. Expression is confined to the oral pole and the oral end of the pharyngeal ectoderm. Expression appears strongest at the oral opening but extends into the pharyngeal ectoderm. Expression is also apparent in scattered cells in the body wall ectoderm, which appear to be neurons based on morphological criteria, namely their position deep within the ectoderm and their basally located nuclei (Figure 5f, Figure 5g) [42]. Later, *Otp *expression is also seen in the tentacle ectoderm (Figure 5h, Figure 5i). Oral expression persists well into polyp stages (Figure 5j, Figure 5k). The probe used for *in situ *hybridization overlaps a substantial portion (< 300 bp) of all three predicted splice variants, so this probe would presumably bind to all three.

### *Rx*

**Figure 6:**
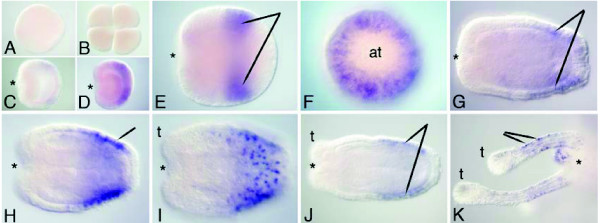
**Expression of *NvRx***. *In situ *hybridization using a 1419 bp probe of *rx*. The asterisks denote the blastopore or oral end. **(a,b) **No expression is detected in the egg or early cleavage stages. **(c,d) **Mid gastrula stages showing expression around the aboral ectoderm. **(e**) Late gastrula stage showing a band of expression towards the aboral pole. **(f) **The same embryo in (e) as viewed from the aboral pole, showing a lack of expression where the future apical tuft (at) will form. **(g-j) **Planula stages, with (i) showing a surface view of the patchy expression. **(k) **Polyp stage, showing a close-up view of expression in the ectoderm of the tentacles (t).

Expression of *Rx *begins before *otp *expression, during mid gastrulation (Figure 6c) *Rx *is initially expressed in the aboral hemisphere (Figure 6d), but by the end of gastrulation, expression becomes refined to a band that encircles the aboral side of the midbody but is excluded from the aboral pole, including the position of the presumptive apical tuft. Within this band, expression is spotty; only a fraction of the cells within the boundaries of the expression domain actually express *Rx*. As with *otp *and *hbn*, these *rx*-expressing cells in the ectoderm have the morphological appearance of neurons (Figure 6f, Figure 6i). *Rx *expression persists in this aboral band throughout planula and polyp stages (Figure 6g-j). During adult polyp stages, a second domain of expression is seen in individual ectodermal cells in the middle of each tentacle (Figure 6).

### *Homeobrain*

**Figure 7:**
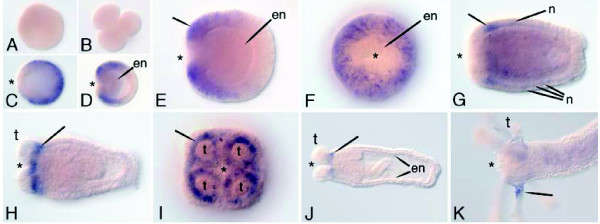
**Expression of *NvHbn***. *In situ *hybridization using a 749 bp probe of *hbn*. The asterisks mark the blastopore or oral end. **(a,b) **Egg and early cleavage stages showing a lack of expression. **(c) **Blastula stage embryo showing expression everywhere except in the blastopore. **(d) **Mid gastrula showing lack of expression in the invaginating endoderm (en). **(e,f) **Lateral and oral views of a late gastrula, stage embryo. **(g) **Planula stage, showing strong expression around the future tentacles (arrow) and in the putative neurons (n) of the mid body. **(h,i) **Early polyp stage showing expression around the base of the tentacles (t). **(j,k) **Older polyp stages, where expression is confined to the base of the tentacles.

*Homeobrain *is first detected in the late blastula, when it is expressed throughout most of the blastoderm except in the region surrounding the blastopore (the presumptive gastrodermis; Figure 7c,d). With the onset of gastrulation, expression becomes excluded from the aboral pole, where the apical tuft of sensory cilia will form (Figure 7d,e). As gastrulation proceeds, expression persists in populations of ectodermal cells around the oral pole and later in those that encircle the base of each developing tentacle (Figure 7g,h). Expression is also seen in individual cells that are scattered throughout the mid body (Figure 7g). These cells have the morphological appearance of neurons. During polyp stages, expression is confined primarily to the ectoderm at the base of the tentacles (Figure 7i-k).

## Discussion

### The functional evolution of hbn, rx and otp

*Homeobrain *was originally identified in *Drosophila *[43], and mapped to a region of chromosome 2 that contained two additional PRD-class homeobox genes, *Orthopedia *and *rx *[44,45]. *Homeobrain *is expressed in the fly embryonic brain and ventral nerve cord [31]. Recently the first lophotrochozan homeobrain-like gene was reported [41], and found to be expressed in a restricted region of the anterior brain. *Hbn *expression has also been reported in the sea urchin, where it is expressed in oral ganglia of the animal pole [46].

*Otp *is associated with neural development in a phylogenetically diverse collection of animals, and this may represent an ancestral role for this homeodomain family. *Orthopedia*-related genes in mouse are involved in brain patterning and development [47-52]. In the hemichordate *Saccoglossus*, *otp *has punctate, ectodermal expression in neural domains of the prosome (proboscis) and mesocome (collar) [53]. In planaria, *orthopedia *is implicated in patterning the branch structure of the brain [54,55]. In the limpet, *otp *is involved in the development of the larval apical sensory organ [56], and in flies it is involved in the developing CNS and hindgut and anal pads [51]. However, a connection to neural development is not obvious in the sea urchin, where *otp *appears to be involved in larval skeletal morphogenesis and the establishment of oral ectodermal cell fate [57-61].

*Rx *(retinal associated homeobox) genes have been extensively studied in many deuterostome taxa including human, mouse, *Xenopus*, chicken, zebrafish, medaka and tunicate [45,62-70]. In all of these organisms, *rx *is involved in brain development and the formation of retinal territories and associated neural structures. In the hemichordate, *rx *is expressed throughout the prosome ectoderm, the most anterior region of this organism, but absent from the most apical pole [53]. *Rx *is expressed more broadly during sea urchin development, with punctate expression in the animal pole and developing gut [46]. A fruit fly *rx *gene has also been identified, and studies suggest that it is necessary for proper brain development, but it is not required for eye development [45,71].

### Alternative splice variants in orthopedia and rx

In addition to a homeodomain and a PRD domain, two additional motifs have been identified in some PRD-like genes. The octapeptide motif [72,73], located towards the N-terminus, is involved in transcriptional repression [74]. The OAR domain, found in *otp*, *aristaless *and *rx *[40,51,67,75] is typically located at the C-terminus and is known to function as a transactivator in *Otp *[40,51].

We have identified alternative transcripts for *Nematostella orthopedia *and *rx*. For rx, some transcripts encode a highly conserved OAR domain and one transcript does not. For *orthopedia*, none of our RACE products nor any ESTs in publicly available databases included the OAR domain. However, a well-conserved OAR domain was identified downstream and in-frame of these sequences in predicted genomic gene models, suggesting that this domain is probably expressed in an as yet unidentified splice variant. The OAR domain has been described as an intramolecular switch, which acts to reduce the affinity of the homeodomain transcription factor for its binding site. In the mouse, ectopically expressed mutant forms of the *Alx3 *and *Cart1 *proteins lacking the OAR domain exhibit increased binding to their DNA targets [76].

Alternative splice variants involving the presence or absence of an OAR domain have also been identified the mouse *prx1 *gene, a member of the PMX family of PRD-class homeobox genes [77]. The carboxy terminus of the *Prx1a *protein includes an activation domain and an OAR domain, whereas the carboxy terminus of *Prx1b *encodes a repression domain and lacks an OAR domain [77,78]. The tissue distribution of both transcripts appears to be similar in mice and humans, but different tissues exhibit pronounced differences in the relative ratios of *prx1a *and *prx1b *[79,80]. It has been hypothesized that the presence of the OAR domain in *prx1a *could render it sensitive to modulation via an unidentified partner protein that interacts with the OAR domain itself. In the absence of this cofactor, the OAR domain masks the activation domain and reduces the affinity of *prx1a *for DNA binding sites. When bound by its co-factor, the activation domain becomes unmasked and the DNA binding affinity increases [76,77,81]. In the case of *Nematostella rx*, the situation may be somewhat simpler than in the mouse *prx1 *gene, because except for the presence or absence of the OAR domain itself, the alternative splice variants encode essentially identical proteins.

### Functional inferences about *Nematostella *otp, rx and hbn based on expression data

In the most famous example of a conserved homeobox cluster, the Hox genes, the spatial ordering of Hox expression territories along the body's main axis and the timing of their onset mirrors the physical ordering of linked Hox genes (although not in all taxa). This correspondence is termed colinearity. However, in animals with dispersed Hox clusters, such as the urochordate *Oikopleura dioica*, some spatial colinearity remains whereas temporal colinearity is absent [82]. This suggests that it is temporal rather than spatial colinearity that is driving the maintenance of these clusters. Although work has been performed in studying temporal colinearity in Hox, ParaHox and NK clusters, the homeobrain cluster could prove to be another supporting example.

In *Nematostella*, *hbn, rx and otp *appear to be expressed in a temporally colinear pattern. *Hbn *is expressed first, in the blastula stages, followed by *rx *at mid gastrulation and finally *otp *in the planula. However, in the case of the *Drosophila homeobrain *cluster, there is no clear evidence of temporal colinearity. Homeobrain is expressed first, in the syncytial blastoderm, then *otp *is expressed slightly before *rx *[31,51,65]. Future studies in other animals will help determine whether temporal colinearity is widely conserved among homeobrain clusters.

Although the expression of *Nematostella hbn*, *rx *and *otp *is consistent with temporal colinearity, it is not consistent with spatial colinearity. The three genes are expressed in non-overlapping domains along the oral-aboral axis; however, these domains do not appear to be related to their position in the cluster. *NvRx *is expressed at the most aboral domain, although expression is not present at the aboral pole, where the apical tuft will form. *NvHbn *is more broadly expressed in oral ectoderm during early embryogenesis and becomes confined to the most oral ectoderm, mainly around the base of the tentacles. Finally, *NvOtp *is also expressed in the oral ectoderm, in the domain that will invaginate and form the pharynx. *NvOtp *and *NvHbn *are both expressed in close proximity to three paralogs of *Otx *(*NvOtxA, B, C*) in the pharyngeal ectoderm and tentacles surrounding the oral pole [30].

In addition to the broad non-overlapping domains, all these genes are also expressed in individual cells throughout the body column. Based on their cell morphology, it appears that these cells may be neurons. Additionally, the expression of *NvOtp *and *NvOtx(A,B,C) *in the oral ectoderm coincides with formation of the oral nerve ring [30,42]. Considering the function of these genes in other animals and the expression patterns seen here, it is likely that *hbn, rx and otp *play some role in neural development in *Nematostella*. However, it is interesting that there is no expression in the apical tuft, another strongly neurogenic region in *Nematostella*.

### The evolutionary history of the homeobrain cluster

The literature on homeobox clusters would suggest that the history of the ANTP class has been qualitatively different from that of the histories of other classes of homeobox genes. The most intensively studied and widely conserved homeobox clusters are all composed of ANTP-class homeobox genes (the Antennapedia complex, the Bithorax complex, the Hox cluster, the ParaHox cluster, the NK cluster and the EGH box cluster). Ultimately, all of these ANTP-class clusters may derive from a single ancestral cluster. For example, the ANTP-C and BX-C of *Drosophila *are clearly derived from a single ancestral Hox cluster that is widely shared by protostomes and deuterostomes, and in simpler form, by cnidarians. The Hox cluster, in turn, appears to have broken off from an ancestral 'mega-Hox' or 'extended Hox' cluster that at one time may have encompassed the Hox cluster, the ParaHox cluster, the EGH cluster and the NK cluster. Over time, the hypothetical ancestral cluster appears to have fragmented, and different remnants of this ancestral cluster may be more highly conserved in different animal lineages [83].

This study is the first to provide evidence that a non-ANTP-class homeobox cluster was conserved over hundreds of millions of years of animal evolution. Clearly, the *hbn*-*rx*-*otp *cluster has been fairly well conserved over the evolutionary history of holometabolous insects. A cluster with the same constituent genes, in the same orientation, spanning a comparable distance is found in representatives of three different orders of insects (Coleoptera, Diptera and Hymenoptera; Figure 4). The cluster also appears to date to the ancestral Protostome, as evidenced by conservation of the three-gene cluster in the limpet *Lottia*, although the order of the genes has changed. In addition, the cluster was also likely to have been present in the cnidarian-bilaterian common ancestor, some 600 million years ago. The cluster in *Nematostella *involves the same closely linked genes in the same order as the inferred ancestral cluster of holometabolous insects, but the *otp *locus has been inverted. Similarly, in four other key taxa (cephalochordate, sea urchin, annelid and placozoan) we find evidence of a partial cluster, in which two of the three genes are linked. Further sequencing and assemblies of the echinoderm and hemichordate genomes will reveal the extent of the *hbn*-*rx*-*otp *cluster in the deuterostome ancestor. The placozoan genomic data suggests that a portion of this cluster (*hbn *and *otp*) dates back even further to the ancestral eumetazoan. However, it remains to be seen whether *rx *was present in this ancestor and lost in the placozoan lineage, or whether *rx *evolved after the split.

Mechanistically, it is easier to envision how a cluster of three closely related genes might remain linked than to envision how three closely related genes, if already dispersed, could independently become so closely juxtaposed in multiple taxa. If the genes arose by tandem duplication, the cluster would have originated as a result of the gene duplication; that is, the starting point would have been a cluster. Subsequently, the cluster could have been maintained over hundreds of millions of years of evolution in multiple animal lineages by stabilizing selection.

Closely linked genes will tend to reside in the same chromosomal territories, and they may come under the influence of shared regulatory elements. For this reason, the proper regulation of linked genes may be related to their physical proximity in the genome. This is the general explanation for why *Hox *genes have remained clustered for hundreds of millions of years in many animals that have been examined. In the case of *Hox *genes, the spatial ordering of *Hox *expression territories along the body's main axis mirrors the physical ordering of linked *Hox *genes along the chromosome. This correspondence is termed colinearity. In the anterior CNS, *rx*, *hbn *and *otp *are expressed in nested territories, which is somewhat reminiscent of *Hox *genes. Future bioinformatics studies could test for conserved regulatory elements within the *hbn*-*rx*-*otp *clusters of fly, honeybee and flour beetle. The functionality of these putative enhancer-binding sites could then be studied experimentally. In addition, the effect of cluster disruption can be examined experimentally in *Drosophila *and in mice, as it has been with Hox genes [84,85]. It may also prove very informative to use evolutionary comparisons of taxa with intact and disrupted clusters to investigate the consequences of cluster disruption, as has recently been carried out for the ParaHox cluster [20].

### The loss of homeobrain in chordates

The *homeobrain *gene appears to have been lost in chordates. We could not identify it in the sequenced genomes of human, mouse, chicken, clawed frog, zebrafish, lancelet or tunicate (Figure 4). However, we identified clear orthologs in two other deuterostomes (sea urchin and hemichordate) and throughout the protostomes and 'basal' metazoans. Thus, this gene may have been truly lost from the genome or it may have become so highly modified that it is no longer recognizable as a *homeobrain *ortholog. The phylogenetic analysis performed here, based on homeodomain sequences, does not strongly suggest another PRD-class gene as a possible ortholog. Unfortunately, we cannot rely on regions outside the 60-amino acid homeodomain to obtain additional phylogenetic signal because there was not sufficient sequence conservation to permit alignments across all genes represented in the phylogeny. The highly conserved 128-amino acid *Paired *domain, present in many PRD-class homeobox genes, is absent from the *homeobrain*, or*thopedia *and *rx *families, among others [29,39,40]. With the present data, our analyses support the hypothesis that *homeobrain *was lost early in the chordate lineage.

### Coding polymorphisms in *Nematostella*

Despite potential stabilizing selection to maintain the cluster, we observed many polymorphisms in the *Nematostella *genes comprising the cluster, including nonsynonymous substitutions in the homeodomain of *rx *that have a patchy distribution in natural populations. The functional role of these polymorphisms awaits future experimental characterization. The presence of few homozygotes for the rare amino acid for each position in our sampling of natural populations is potential evidence for a functional difference between alleles.

## Conclusions

A PRD-class homeobox cluster comprising *homeobrain*, or*thopedia *and *rax *is widely conserved in animals and thus represents an ancient gene cluster dating to early in metazoan evolution. Very similar clusters comprising these three genes are present in *Nematostella*, in diverse protostomes, and potentially in non-chordate deuterostomes. Chordates appear to have lost one member (*homeobrain*) and the linkage between the other two genes, *rx *and *orthopedia*, has dissolved in the diverse vertebrates we examined.

## Competing interests

The authors declare that they have no competing interests.

## Authors' contributions

MEM performed the majority of the gene mapping analysis, participated in the phylogenetic analysis and contributed to drafting the manuscript. KP performed the *in situ *hybridization studies. AMR performed the population genetic studies of rx and participated in the gene mapping studies and phylogenetic analysis. MQM participated in the design of the study and oversaw the analysis of the gene expression data. JRF participated in the design of the study and the phylogenetic analysis, and contributed to drafting the manuscript. All authors read and approved the final manuscript.

## Supplementary Material

Additional file 1 **OTP annotation**. Alignment of *Orthopedia *transcripts against the assembled genome. Three *otp *transcripts were mapped against scaffold_62 of the publicly available *Nematostella *genome assembly. The position relative to the scaffold is indicated to the right of the nucleotide sequence. For transcripts 1-3, identity to the genomic sequence is indicated with a full stop (.). Long introns have been truncated for clarity. Polymorphic positions are highlighted in black. We reconstructed one *otp *transcript (1) by conceptually splicing overlapping 3' and 5' RACE fragments. This transcript is 1045 nucleotides long and it maps between positions 812065 and 802467 of the scaffold. Another *otp *transcript (2) was identified among the ESTs sequenced as part of the *Nematostella *genome project (jgi|Nemev1|205678|fgenesh1_pg.scaffold_62000087). This transcript maps between positions 790736 and 812044 of the scaffold. A third *Otp *transcript (3) had been previously deposited in the EST database at NCBI [86]; GenBank accession DV090169). This transcript is only 616 nucleotides in length and it appears to be truncated at both ends. The predicted amino acid sequences are shown beneath the nucleotide sequences. Three conserved domains are indicated in bold type; the octapeptide (HSIVGILN), the 60 amino acid homeodomain and the 16 amino acid OAR domain. The OAR domain is downstream and in frame with the homeodomain, but the boxed amino acids are not encoded by any of the three *otp *transcripts we recovered. Click here for file

Additional file 2 **RX annotation**. Alignment of *rx *transcripts against the assembled genome. We reconstructed one *rx *transcript (1) by conceptually splicing overlapping 3' and 5' RACE fragments (RACE). We also identified two *rx *sequences among the 150,000 ESTs generated by the Joint Genome Institute *Nematostella *sequencing project (2: 2664141-1, 3: 2664141-2) and two *rx *ESTs that were previously deposited at NCBI (4: CAGN10625, 5:CV088198). The RACE product spans nucleotides 785,552 to 790,345 of scaffold_62 in the Joint Genome Institute *Nematostella *genome assembly. Location relative to the scaffold is indicated to the right of the nucleotide sequence. The long second intron (3713 nucleotides in length) has been truncated for clarity. Polymorphic nucleotides are highlighted in black. Corresponding polymorphic amino acids are boxed. The predicted amino sequence is shown below the nucleotide sequence. Three conserved motifs are shown in bold type: the octapeptide (HSIDAILG), the 60-amino acid homeodomain and the 16-amino acid OAR motif. There are two non-silent polymorphisms within the homeodomain (K/R at position 52 and E/Q at position 59 (see Figure 5 for geographic distribution). The EST CV088198 (5) does not encode the complete OAR motif. It encodes a predicted protein (ending in a phenylalanine) that is 24 residues shorter than the predicted protein encoded by the other transcripts. Click here for file

Additional file 3 **RX polymorphism map**. Geographic distribution of *rx *polymorphisms. In total, 95 individual animals were successfully genotyped at each of the two polymorphic positions in the *Rx *homeodomain from 24 estuaries. Collection sites were as follows: 1, Spurwink River, ME; 2, Odiorne Point, NH; 3, Rye Harbor, NH; 4, Wallis Sands, NH; 5, Old Town Hill, MA; 6, Crane Reserve, MA; 7, Neponset River, MA; 8, Pocasset River, MA; 9, Sippewissett Marsh, MA; 10, Clinton, CT; 11, Kingsport, Nova Scotia; 12, Halifax, NS; 13, Meadowlands, NJ; 14, Rhodes River, MD: 15, Baruch, SC; 16, San Juan Island, WA; 17, Willapa Bay, WA; 18, Coos Bay, OR; 19, Humboldt, CA; 20, Bodega Bay, CA; 21, Tomales Bay, CA; 22, Fort Gillkicker Lagoon, UK; 23, Salterns, UK; 24, Half Moon Lagoon, UK. The overall genotypic frequencies are: position 52: KK = 71.58%, KR = 26.16%, RR = 2.11%; position 59: QQ = 92.55%, QE = 7.45%, EE = 0.00%. Click here for file

Additional file 4 **HBN annotation**. Annotated *Nematostella Homeobrain *locus. We reconstructed one *hbn *transcript (1) by conceptually splicing overlapping 3' and 5' RACE fragments (RACE). We also identified three *Hb *ESTs that were previously deposited at NCBI (2: DV0879878, 3: DV084683; 4: DV086666). The transcript obtained by RACE is 1139 nucleotides long and comprises three exons, which collectively span nucleotide positions 777,772 to 782,115 of scaffold_62 in the Joint Genome Institute *Nematostella *genome assembly. The position relative to the scaffold is indicated to the right of the nucleotide sequence. The predicted amino acids are shown below the nucleotides that encode them. Polymorphic nucleotides are highlighted in black. Corresponding polymorphic amino acids are boxed. Long introns have been truncated for clarity. Three conserved protein motifs are shown in bold type; the octapeptide (YTIDMILG), the 60-amino acid homeodomain and the 16-amino acid OAR domain. Click here for file
