# Supplementary material for: A conserved cluster of three PRD-class homeobox genes (homeobrain, rx and orthopedia) in the Cnidaria and Protostomia
Source: EvoDevo. 2010 Jul 5;1:3. doi: 10.1186/2041-9139-1-3 (PMC2938728; doi:10.1186/2041-9139-1-3)
Supplement: Additional file 1 — OTP annotation. Alignment of Orthopedia transcripts against the assembled genome. Three otp transcripts were mapped against scaffold_62 of the publicly available Nematostella genome assembly. The position relative to the scaffold is indicated to the right of the nucleotide sequence. For transcripts 1-3, identity to the genomic sequence is indicated with a full stop (.). Long introns have been truncated for clarity. Polymorphic positions are highlighted in black. We reconstructed one otp transcript (1) by conceptually splicing overlapping 3' and 5' RACE fragments. This transcript is 1045 nucleotides long and it maps between positions 812065 and 802467 of the scaffold. Another otp transcript (2) was identified among the ESTs sequenced as part of the Nematostella genome project (jgi|Nemev1|205678|fgenesh1_pg.scaffold_62000087). This transcript maps between positions 790736 and 812044 of the scaffold. A third Otp transcript (3) had been previously deposited in the EST database at NCBI [86]; GenBank accession DV090169). This transcript is only 616 nucleotides in length and it appears to be truncated at both ends. The predicted amino acid sequences are shown beneath the nucleotide sequences. Three conserved domains are indicated in bold type; the octapeptide (HSIVGILN), the 60 amino acid homeodomain and the 16 amino acid OAR domain. The OAR domain is downstream and in frame with the homeodomain, but the boxed amino acids are not encoded by any of the three otp transcripts we recovered. [file 2041-9139-1-3-S1.PDF]

```
      GCAATAAGCAGTTCTACAAAGAACACCGAAATAGTGCAACGATCAGAAGGTTATTATGGAGCTCTCGGGCGACACACACG 812020
                                                                  M  E  L  S  G  D  T  H
1                                            ....................................
2                                                                     ........................
      GACCGAGAGGGAGCAGTATTGATTCTCATGAAGAAAATTCGACAAGTTCCTTGCTTGAGCTGGTGCCAAGCTTAGAACAC 811940
      G  P  R  G  S  S  I  D  S  H  E  E  N  S  T  S  S  L  L  E  L  V  P  S  L  E  H
1,2   ................................................................................
      TCCATCGTGGGAATACTTAATCATAATGGGGAAATCAATGAACACGATTCTGGTAGAGTAATTGAAGTTGCTAGGGTATC 811860
       S  I  V  G  I  L  N  H  N  G  E  I  N  E  H  D  S
1,2   ........................................................
3           ..........................................
      AAATCTTTGCCAGTTTTAACTTATCAACCATTAACCAGGACTTATAAAAATAAACGCTCTAATGATTTACACTGAAACTG 811780
      GCTCTAATTGAAATCACTTCTCTTCCTTATACAGATAATTCAAAAGCAGTTAGCATTGACGGCAAGTCTTCTCCGGGTGA 811700
                                                  D  N  S  K  A  V  S  I  D  G  K  S  S  P  G  D
1,2,3                                             ..............................................
      CTTAAAGAAAAACCTGGACGATCAGGACGACGACAGAAAGTCCACGGACAGCCAGGCAGCCAAACAGAAGCGCCACCGAA 811620
       L  K  K  N  L  D  D  Q  D  D  D  R  K  S  T  D  S  Q  A  A  K  Q  K  R  H  R
1,2,3 ................................................................................
      CGCGATTCACTCCCGCTCAATTGAACGAGCTCGAGCGATGTTTCGCCAGGACTCACTATCCGGATGTGTTCATGCGAGAA 811540
      T  R  F  T  P  A  Q  L  N  E  L  E  R  C  F  A  R  T  H  Y  P  D  V  F  M  R  E
1,2,3 ................................................................................
      GAGCTGGCAGCGCGAATAGGACTCACGGAGTCCAGAGTGCAG~~~~~~~~~~~GTTTGGTTCCAGAACCGCCGCGCCAAG 803940
       E  L  A  A  R  I  G  L  T  E  S  R  V  Q           V  W  F  Q  N  R  R  A  K
1,2,3 ......................................... [7440 nts]..........................
      TGGAAGAAGCGTAAGAAGACCGCGTCCATTTTGCGTCCGCCCGCACCCATTCTGCCATCCCACATGGCACAGGGTTACAA 803860
       W  K  K  R  K  K  T  A  S  I  L  R  P  P  A  P  I  L  P  S  H  M  A  Q  G  Y  N
1,2,3 ................................................................................
      CGCCGGCCCGATTGGTGACACTCTCTGCACATTCCACAACGATCACCGCTGGCCACCTACGGTCACGGCCACCATGCCGA 803780
        A  G  P  I  G  D  T  L  C  T  F  H  N  D  H  R  W  P  P  T  V  T  A  T  M  P
1,2,3 ................................................................................
      CAATGGCTCCAGGCCCCTCTCCATCACTGCCCCTGTCGCCCCCACATCACCCGTTCACGCAGGCGCACGAGGTTTTACAG 803700
      T  M  A  P  G  P  S  P  S  L  P  L  S  P  P  H  H  P  F  T  Q  A  H  E  V  L  Q
1,2,3 ................................................................................
      CAATCCTACCCACTCCAGTCCCCCCTTCAGCGCTCTCACCTTTCAATGGCTATCCAGCAGCAGAGCAGTCAGCAATCGCA 803620
       Q  S  Y  P  L  Q  S  P  L  Q  R  S  H  L  S  M  A  I  Q  Q  Q  S  S  Q  Q  S  Q
1,2   ................................................................................
3     ............................................C........
      GCAGAACTACCAACAGCCGTACGTGTCGAGAGAAATGACCATATCCTCGTCGCCCACGCTCCAGGACATCCAGTGCAACA 803540
        Q  N  Y  Q  Q  P  Y  V  S  R  E  M  T  I  S  S  S  P  T  L  Q  D  I  Q  C  N
1,2   ................................................................................
      TCAACGGCGGTGAGCAATGGCGGGGGACAAGCATCGCGAGCTTGCGCAGAAAAGCACTCGAGCACCAGGCCACGCTCGTG 803460
      I  N  G  [G  E  Q  W  R  G  T  S  I  A  S  L  R  R  K  A  L  E  H  Q  A  T  L  V]
1,2   .........
      TACAGCAGATAGAGGCAAACCTAGGGGAGGGATGGGACAGGCATTAAGCAGGGGAACGAGGGTGTAGAAAGGCACTCAAA 803380
      [Y  S  R  *]
      ~~~~~~~~~~~~~~~~~~~~~~~~~~~~~~~~~~~720 nucleotides~~~~~~~~~~~~~~~~~~~~~~~~~~~~~~
      GTTGTTTTAGGTGTTTCAACCAGAGGGCTATGGACTCGATTCTGACCCCCTGCAGCGGCGGCAAGCCCTGGATTTAAGTA 802580
                 V  S  T  R  G  L  W  T  R  F  *
1                ................................................................
      AAATACTCATAGATGGCTATAAATATCATAAACCAATTCAATTGATTTATCTGTAATATAGCGAAGCGAATTGTACAATG 802500
1     ................................................................................
      CGTTATTCCAGAAAAAATATCCCCAAACTTCTGCCGTATGGATGGCACAAAAGAAGTAGTATCCACTTGGCCAAACGGAAG 802420
1     ......................T...........
      TCTGTCTCTATAGGGTACCCCGAGTGGCACATTGACGGAGTTGTCACTCTTGGGTTCGATTCTCGGTTAGACGGAGCGAC 802340
                    G  Y  P  E  W  H  I  D  G  V  V  T  L  G  F  D  S  R  L  D  G  A  T
2                   ..............................................................
      CCAAAAGCTTTCACAATTTTTCAGACTACAAGCAACCTATATTCTGTTGTATCCCTTGACATTAGTGAAGAACAAGGAGC 802260
        Q  K  L  S  Q  F  F  R  L  Q  A  T  Y  I  L  L  Y  P  L  T  L  V  K  N  K  E
2     ................................................................................
      ATTACACATGCCAAATGGTGATACTGAAGGTGAACGAGGTGAGGATAAAGCGAACCCAAAATACTCATTATTTTTCATAT 802180
      H  Y  T  C  Q  M  V  I  L  K  V  N  E
2     ..................................
      ~~~~~~~~~~~~~~~~~~~~~~~~~~~~~~~~~~11364 nucleotides~~~~~~~~~~~~~~~~~~~~~~~~~~~~~
      TATTACAGGATGAATCGAGCCTTGGTAGAATGTGGGTTTACAATGGTAGCCAGCAAATCGGATTAAGATATCCTCGTTAA 790736
              D  E  S  S  L  G  R  M  W  V  Y  N  G  S  Q  Q  I  G  L  R  Y  P  R  *
              ...................................................................
```
