# Supplementary material for: A conserved cluster of three PRD-class homeobox genes (homeobrain, rx and orthopedia) in the Cnidaria and Protostomia
Source: EvoDevo. 2010 Jul 5;1:3. doi: 10.1186/2041-9139-1-3 (PMC2938728; doi:10.1186/2041-9139-1-3)
Supplement: Additional file 2 — RX annotation. Alignment of rx transcripts against the assembled genome. We reconstructed one rx transcript (1) by conceptually splicing overlapping 3' and 5' RACE fragments (RACE). We also identified two rx sequences among the 150,000 ESTs generated by the Joint Genome Institute Nematostella sequencing project (2: 2664141-1, 3: 2664141-2) and two rx ESTs that were previously deposited at NCBI (4: CAGN10625, 5:CV088198). The RACE product spans nucleotides 785,552 to 790,345 of scaffold_62 in the Joint Genome Institute Nematostella genome assembly. Location relative to the scaffold is indicated to the right of the nucleotide sequence. The long second intron (3713 nucleotides in length) has been truncated for clarity. Polymorphic nucleotides are highlighted in black. Corresponding polymorphic amino acids are boxed. The predicted amino sequence is shown below the nucleotide sequence. Three conserved motifs are shown in bold type: the octapeptide (HSIDAILG), the 60-amino acid homeodomain and the 16-amino acid OAR motif. There are two non-silent polymorphisms within the homeodomain (K/R at position 52 and E/Q at position 59 (see Figure 5 for geographic distribution). The EST CV088198 (5) does not encode the complete OAR motif. It encodes a predicted protein (ending in a phenylalanine) that is 24 residues shorter than the predicted protein encoded by the other transcripts. [file 2041-9139-1-3-S2.PDF]

```
         CATCATGTATACTCTTAATAGCCTCATATTAAGGGGCCATACCGTGAGCAGTTTAGTCCACACATCACATCGCGCAAGTA 790320
1                                                              ..........................
2             .....................................................T.....................
3                                                                                 ........
4                                                                             ............
         TGAGCGACCATAAAAACATAGAAGATCGAAAGAGTAGGGAAGAGGAGATAGTCAGTCTGAAGCCACAAAACATCCACTCA 790240
         M  S  D  H  K  N  I  E  D  R  K  S  R  E  E  E  I  V  S  L  K  P  Q  N  I  H  S
1-4      ................................................................................
         ATCGATGCCATATTAGGCAAGAAAGAGCCCGCGAGAAGACCAGAACAAGAGCGAAAGACTATCAACGACTCCAACCCAAG 790160
          I  D  A  I  L  G  K  K  E  P  A  R  R  P  E  Q  E  R  K  T  I  N  D  S  N  P  R
1-4      ................................................................................
         ACTAGAGGAAGACGATATCAATTCAGACAATGATTTGAGCTCCAACGATGGTAAATCAGGTATAACCGCATCATTAACCT 790080
           L  E  E  D  D  I  N  S  D  N  D  L  S  S  N  D  G  K  S
1-4      .........................................................
         GTTGTGGGGTATTCCAGGATAGACCCTGAGTGTAATTTGATACTAATATTGTTCGTGCTGTATCTATTAGACGAAGCCGA 790000
                                                                               D  E  A  D
1-4                                                                           ...........
         CGGTGACTCGTCAAAGAAAAAGCTCAGAAGAAACCGTACCACCTTCACAACGTTTCAGTTACACGAACTCGAGCGAGCCT 789920
           G  D  S  S  K  K  K  L  R  R  N  R  T  T  F  T  T  F  Q  L  H  E  L  E  R  A
1-4      ................................................................................
         TCGAGAAATCCCACTATCCAGACGTGTACACAAGAGAAGAGTTGGCGCTAAAAATAAGCCTCCCTGAAGTTAGGGTACAG 789840
         F  E  K  S  H  Y  P  D  V  Y  T  R  E  E  L  A  L  K  I  S  L  P  E  V  R  V  Q
1-4      ................................................................................
5                      ..................................................................

         ~~~~~~~~~~~~~~~~~~~~~~~~~~~~~~~~~~~3600 nucleotides~~~~~~~~~~~~~~~~~~~~~~~~~~~~~
         TTATTAACCAATTTCCCTTCTAATTTACCACAGGTGTGGTTCCAGAACAAAAGAGCAAAATGGCGTCGAGAGGAGAAGAT 786080
                                           V  W  F  Q  N K/R R  A  K  W  R  R E/Q  E  K  M
1,2                                       ...............................................
3,4                                       ................G...................C..........
5                                         ................G...................C........G.
         GGAAATGGCGAGCCTGCAAGACCTCCCCTCCCCGTCCAGAACAAGCGGATTCGGTAGTCTGACGTTCTCAGATATGTGGA 786000
           E  M  A  S  L  Q  D  L  P  S  P  S  R T/I S  G  F G/C S  L  T  F  S D/Y M  W
1,2      ...............................................................A................
3,4      ................................................................................
5        ......................................N..G..........T...........T.......T.........
         AAAACCCGCTCACACTGACTGGCCCATACGGGGCTTTGTTCCCAAGGTCCGGGGGATCCCCGCCACCGGGTATCGGGTAT 785920
         K  N  P  L  T  L  T  G  P  Y  G  A  L  F  P  R  S  G  G  S  P  P  P  G  I  G  Y
1-4      ................................................................................
5        ......................G....................TC...................................
         TACCCGCCATATGGCCATTCGGCGCTCGCCGCTTTCTCGTATTTACCCCAAGTCGGATGTGGTTCTGCGATGGCAGAGAT 785840
          Y  P  P  Y  G  H  S  A  L  A  A  F  S  Y  L  P  Q  V  G C/S G  S  A  M  A  E  M
1,2      ................................................................................
3,4      ........................................................G.......................
5        .....................................................G..GA.........G....T.T.....
         GACAATACCCCTAAGCTCCCCCTCGAATTCAACCGACGAGCGATCGTCTAGTATTGCTACATTAAGGCTCAAGGCCAAAG 785760
           T  I  P  L  S  S  P  S  N  S  T  D  E  R  S  S  S  I  A  T  L  R  L  K  A  K
1,2      ................................................................................
3,4      ................C...............................................................
5        .............CC.G.............C......C.T.......
         AACATCTAGAAAGCATGGAAGAGATGAAAGTCTGTCTGCCTGAGGGCTTTAACTAAACACCATCCCGGGCACTAATACG 785680
         E  H  L  E  S  M  G  R  D  E  S  L  S  A  *     F  K  *
1,3,4    ................................................................................
2        ............A...................................................................
5                                                          ....G
         TGGCTACTCGTATCAGATTTGCCCGAAATATCGCAAGATTATCTGCCGATAATCGGGATTATCTTCCAAAGACGTAATGG 785600
1,2      ................................................................................
3        .........................................C.C....................................
4,5      .........................................C.C.....
         AAAACATTAGAACTGTTAAAGATAGATTTGAAAAAAAAAAAAAAAAAACGGTGGGATATTCTTTTATTTTATTCTTTGTAC 785520
1        .........T.....................................
2        .........T................-..................
3        ..............................
```
