# Supplementary figures and images for: A conserved cluster of three PRD-class homeobox genes (homeobrain, rx and orthopedia) in the Cnidaria and Protostomia
Source: EvoDevo. 2010 Jul 5;1:3. doi: 10.1186/2041-9139-1-3 (PMC2938728; doi:10.1186/2041-9139-1-3)

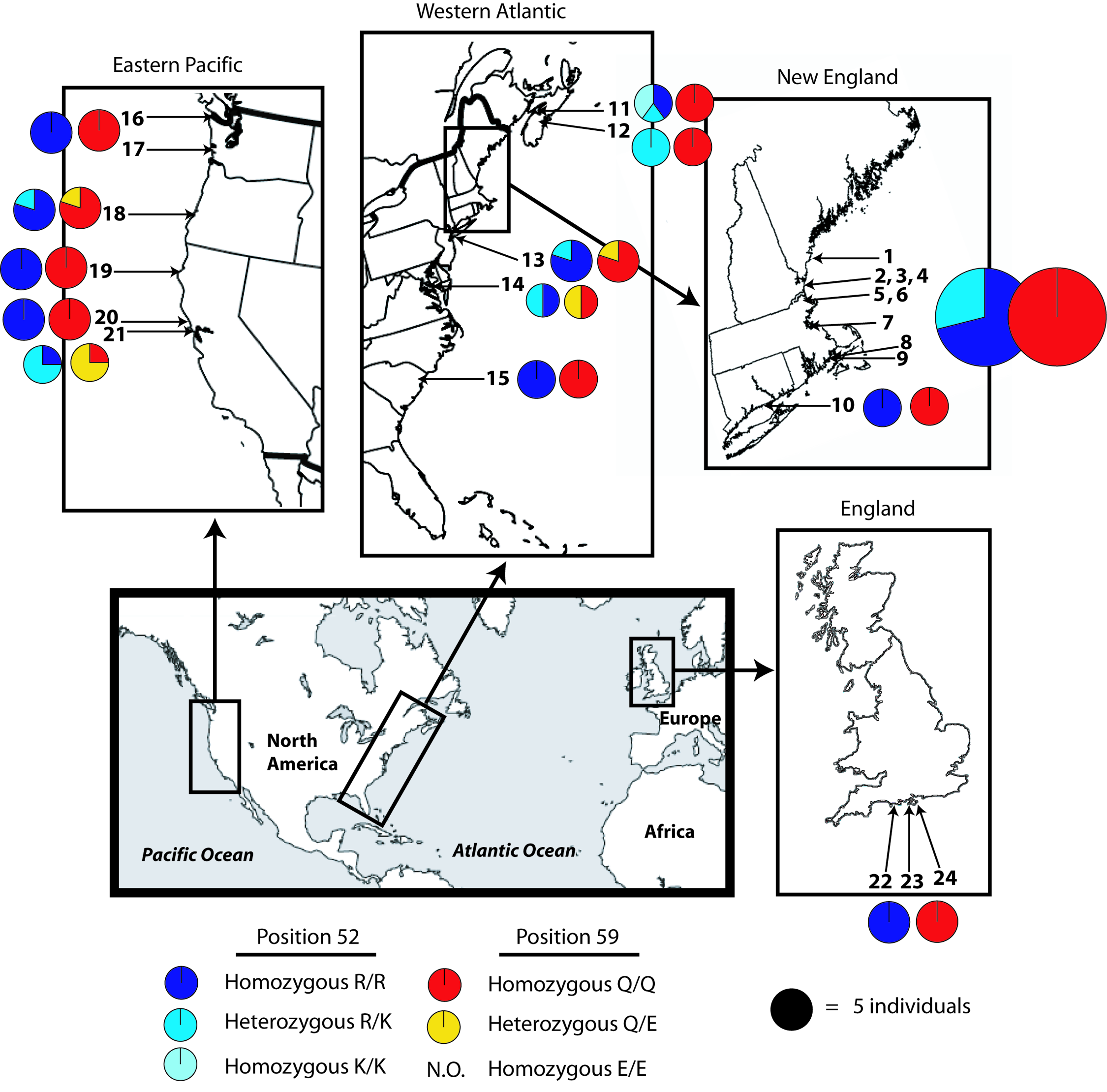

Supplement: Additional file 3 — RX polymorphism map. Geographic distribution of rx polymorphisms. In total, 95 individual animals were successfully genotyped at each of the two polymorphic positions in the Rx homeodomain from 24 estuaries. Collection sites were as follows: 1, Spurwink River, ME; 2, Odiorne Point, NH; 3, Rye Harbor, NH; 4, Wallis Sands, NH; 5, Old Town Hill, MA; 6, Crane Reserve, MA; 7, Neponset River, MA; 8, Pocasset River, MA; 9, Sippewissett Marsh, MA; 10, Clinton, CT; 11, Kingsport, Nova Scotia; 12, Halifax, NS; 13, Meadowlands, NJ; 14, Rhodes River, MD: 15, Baruch, SC; 16, San Juan Island, WA; 17, Willapa Bay, WA; 18, Coos Bay, OR; 19, Humboldt, CA; 20, Bodega Bay, CA; 21, Tomales Bay, CA; 22, Fort Gillkicker Lagoon, UK; 23, Salterns, UK; 24, Half Moon Lagoon, UK. The overall genotypic frequencies are: position 52: KK = 71.58%, KR = 26.16%, RR = 2.11%; position 59: QQ = 92.55%, QE = 7.45%, EE = 0.00%. [file 2041-9139-1-3-S3.TIFF]
