# Supplementary material for: A conserved cluster of three PRD-class homeobox genes (homeobrain, rx and orthopedia) in the Cnidaria and Protostomia
Source: EvoDevo. 2010 Jul 5;1:3. doi: 10.1186/2041-9139-1-3 (PMC2938728; doi:10.1186/2041-9139-1-3)
Supplement: Additional file 4 — HBN annotation. Annotated Nematostella Homeobrain locus. We reconstructed one hbn transcript (1) by conceptually splicing overlapping 3' and 5' RACE fragments (RACE). We also identified three Hb ESTs that were previously deposited at NCBI (2: DV0879878, 3: DV084683; 4: DV086666). The transcript obtained by RACE is 1139 nucleotides long and comprises three exons, which collectively span nucleotide positions 777,772 to 782,115 of scaffold_62 in the Joint Genome Institute Nematostella genome assembly. The position relative to the scaffold is indicated to the right of the nucleotide sequence. The predicted amino acids are shown below the nucleotides that encode them. Polymorphic nucleotides are highlighted in black. Corresponding polymorphic amino acids are boxed. Long introns have been truncated for clarity. Three conserved protein motifs are shown in bold type; the octapeptide (YTIDMILG), the 60-amino acid homeodomain and the 16-amino acid OAR domain. [file 2041-9139-1-3-S4.PDF]

```
         AAAGAACGCACGCAGTTACTTCTAGCAGTACAGGGCGATGAATCGCTATCAAAGGCCAAGCATGGTGTACTCTGAATCTG 777840
                                                      M  N  R  Y  Q  R  P  S  M  V  Y  S  E  S
1                    ...........................................................................
         AGGACAAAAAGACCAGCCAAATGCACAGACATTCCGTTGAGTCTGCTCTCATCCAAGCCTCAAATAAAGGTTATACTATA 777920
          E  D  K  K  T  S  Q  M  H  R  H  S  V  E  S  A  L  I  Q  A  S  N  K  G  Y  T  I
1        ................................................................................
         GATATGATTTTGGGGAATCGAGAGAATTCGACGGCAAAGCGTGAAGAGAAACGACCGGTTGAGTACAACCCAGTAGGCCA 778000
          D  M  I  L  G  N  R  E  N  S  T  A  K  R  E  E  K  R  P  V  E  Y  N  P  V  G  Q
1        ................................................................................
         AGGAGATACCAGCGACACTGGTGAGTTCGGCTCGCCACTTCTCATACAATCATTGATTTACAAATAACACAGCTAATCTT 778080
           G  D  T  S  D  T
1        ....................
         ~~~~~~~~~~~~~~~~~~~~~~~~~~~~~~~~560 nucleotides~~~~~~~~~~~~~~~~~~~~~~~~~~~~~~~~~
         GTACGATCAAGCAAACAGGTCTAAAAATTACTCTTATTCAAACAGAGGAACAAGAAAAAGTGATGAAGGACGATGGCAGT 778720
                                                                E  Q  E  K  V  M  K  D  D  G  S
1        ................................................................................
         GGCGAAGAGAGCGCAGGTGAGGATGGAAAGCCGAGGAAAGTCAGGCGAAGTCGAACTACGTTCACGACATACCAACTTCA 778800
          G  E  E  S  A  G  E  D  G  K  P  R  K  V  R  R  S  R  T  T  F  T  T  Y  Q  L  H
1        ................................................................................
         CCAACTCGAGCGCGCGTTTGAGAAAACGCAATACCCCGATGTGTTCACGAGAGAGGAACTTGCCCTTCGGCTGGACTTGA 778880
           Q  L  E  R  A  F  E  K  T  Q  Y  P  D  V  F  T  R  E  E  L  A  L  R  L  D  L
1        ................................................................................
2        ................................................................................
         GCGAGGCAAGAGTACAGGT~~~~~~~AGGTCTGGTTTCAAAACCGCAGAGCCAAGTGGCGTAAACGAGAAAAGGCCCTCG 781520
         S  E  A  R  V  Q [2480 nts] V  W  F  Q  N  R  R  A  K  W  R  K  R  E  K  A  L
1,2      .................
1,2,3                                ....................................................
         GACGAGAGAGCCCCAACTTTATGTACCCAGGACACGGCGGAGAGTACCGTGGGGTCCCTACTGAATTACCGCCCCTTCAT 781600
         G  R  E  S  P  N  F  M  Y  P  G  H  G  G  E  Y  R  G  V  P  T  E  L  P  P  L  H
1,3,4    ................................................................................
2        .................
         ACTGCATACCAACCCGTGCCCCCTTCGTTCATGGACCGGTGGAACCCCCACGTCCCTGCTCTCAGCCTCATCTCTCCATA 781680
          T  A  Y  Q  P  V  P  P  S  F M/T D  R  W  N  P  H  V  P  A  L  S  L  I  S  P  Y
1,4      ................................................................................
3        ...............................C................................................
         CGCCACAGCACCTCCCCAATATGGTCCCATGGGGCCACATATTGGCGCTCCTGGATACTACTCGCCACACCTCCTGAGGC 781760
           A  T  A  P  P  Q  Y  G  P  M  G  P  H  I  G  A  P  G  Y  Y  S  P  H  L  L  R
1,3,4    ................................................................................
2                                                                     ..................
         ACTATATCCATCCTGCTATGGTCCCTTATCTAAAAGGGCCCCTGAGTCCACCCCAGGGACATGATATGAGGAGGACAAGC 781840
         H  Y  I  H  P  A  M  V  P  Y  L  K  G  P  L  S  P  P  Q  G  H  D  M  R  R  T  S
1,3,4    ................................................................................
2        ............................................................................C...
         ATCGAGGATCTTCGCCACAAAGCGAAGAAACATTCCGCCTCAATCTCCAGCCCGGAGGAGGGGAATTCGAGCTCATAATG 781920
          I E/G D  L  R  H  K  A  K  K  H  S  A  S  I  S  S  P  E  E  G  N  S S/G S  *
1,2,3    ................................................................................
4        ....G...............................................................G...........
         CAAGCAATACAATCAATATTATTACCCTTTCATGTGAGAGGGCTTGTATATAGTATTTGTAACATATACATATATATTTT 782000
1,3,4    ................................................................................
                                                                                   AT
                                                                                    |
2        ................................................................................
         TATGACAATAGTTTTCTATAATGCAATTTAAACAGTGATGCTTTATATCCAATTAAAGCAAATTGCAATGTAAAACTATG 782080
1,4      ................................................................................
2        ....................C..............T.............................................
3        ...................................G......................
         AATTCGGTATTTTAATATTAAAACACGGTTCAGACCTTACCAACACCGGTATTTTCACAACGCAATTTTTTGCGCAATTT 782160
4        ................................................................................
1        ...................................
2        ........................G.................
         TCCCTAAGGCTATTTCGGAGTCGTAATCCCTAACAAAATACCTCCTACATAAAGAAACACAATTTAAATATAAACGCACG 782240
4        .......................................-.-----..----.............................
         TGCCTTTTTTGTCCATTTATGCATAAAGAAATTTCAGATTCCAGGGAAAAATGCAAAACAAATTAAGCTTCTTTACCTAT 782320
4        ..........
```
